# The importance of the intestinal microbiota in humans and dogs in the neonatal period

**DOI:** 10.1590/1984-3143-AR2023-0082

**Published:** 2023-11-10

**Authors:** Samara Beretta, Maricy Apparicio, Gilson Hélio Toniollo, Marita Vedovelli Cardozo

**Affiliations:** 1 Departamento de Patologia, Reprodução e Saúde Única, Faculdade de Ciências Agrárias e Veterinárias (FCAV), Universidade Estadual Paulista (UNESP), Jaboticabal, SP, Brasil; 2 Departamento de Cirurgia Veterinária e Reprodução Animal, Faculdade de Medicina Veterinária e Zootecnia (FMVZ), Universidade Estadual Paulista (UNESP), Botucatu, SP, Brasil; 3 Laboratório de Fisiologia de Microorganismos, Departamento de Ciências Biomédicas e Saúde, Universidade do Estado de Minas Gerais (UEMG), Passos, MG, Brasil

**Keywords:** gut microbiome, dysbiosis, gut-brain axis, vaginal seeding

## Abstract

The neonatal period represents a critical stage for the establishment and development of the gut microbiota, which profoundly influences the future health trajectory of individuals. This review examines the importance of intestinal microbiota in humans and dogs, aiming to elucidate the distinct characteristics and variations in the composition between these two species. In humans, the intestinal microbiota contributes to several crucial physiological processes, including digestion, nutrient absorption, immune system development, and modulation of host metabolism. Dysbiosis, an imbalance or disruption of the gut microbial community, has been linked to various disorders, such as inflammatory bowel disease, obesity, and even neurological conditions. Furthermore, recent research has unveiled the profound influence of the gut-brain axis, emphasizing the bidirectional communication between the gut microbiota and the central nervous system, impacting cognitive function and mental health. Similarly, alterations in the canine intestinal microbiota have been associated with gastrointestinal disorders, including chronic enteropathy, such as inflammatory bowel disease, food allergies, and ulcerative histiocytic colitis. However, our understanding of the intricacies and functional significance of the intestinal microbiota in dogs remains limited. Understanding the complex dynamics of the intestinal microbiota in both humans and dogs is crucial for devising effective strategies to promote health and manage disease. Moreover, exploring the similarities and differences in the gut microbial composition between these two species can facilitate translational research, potentially leading to innovative therapeutic interventions and strategies to enhance the well-being of both humans and dogs.

## Introduction

The neonatal period in dogs corresponds to the first two to four weeks of life (Grundy, 2006; Vannucchi et al., 2012; Mila et al., 2017a; Vassalo et al., 2015). It is a challenging period (Konde et al., 2015; Chastant-Maillard et al., 2017) characterized by high rates of morbidity (Konde et al., 2015) and mortality. The main causes of death include stillbirth, maternal and environmental factors, as well as infections (Indrebø et al., 2007; Lourenço, 2015). Infectious diseases, particularly those caused by bacteria, are the second most common cause of death during this period (Meloni et al., 2014; Münnich and Küchenmeister, 2014).

The mammalian intestinal tract harbors numerous microbial populations and plays crucial roles in host health, such as providing nutritional substrates, modulating the immune system, and aiding in defense against intestinal pathogens (Spor et al., 2011; Pitlik and Koren, 2017). Studies indicate that early intestinal colonization in human newborns starts with exposure to maternal vaginal and fecal microbiota, which is essential for normal intestinal development and function (Insoft et al., 1996; McCracken and Lorenz, 2001; Dominguez-Bello et al., 2010; Torrazza and Neu, 2011). Moreover, the mode of delivery, whether cesarean or vaginal, may influence the incidence of neonatal sepsis. Cesarean section has been shown to affect the intestinal microbiota and neonatal immunity in both humans and mice, thereby impacting the risk of bacterial infection (Shao et al., 2019; Zachariassen et al., 2019). In dogs, differences have been observed in the microbiota of puppies born vaginally versus those delivered by cesarean section, with higher rates of growth and weight gain in the former (Zakošek Pipan et al., 2020).

Alterations in gut microbiota have been associated with various gastrointestinal and systemic diseases, highlighting the potential utility of studying intestinal microbiota in diagnostic procedures and therapies (Garrigues et al., 2023).

While there is extensive research on the intestinal microbiota in humans, our understanding of the development of intestinal microbiota in puppies is limited. Gaining insight into the early evolution of the canine microbiota is crucial for improving the short- and long-term health and well-being of dogs. Therefore, comprehending how dogs can serve as experimental models for human studies is highly relevant, as it would facilitate the design and execution of new studies that provide dual benefits for both canine and human health.

The objective of this work is to review the literature regarding the influence of the intestinal microbiota on neonatal and pediatric development in both humans and dogs, and to explore the various research possibilities involving dogs as an experimental model for applications in human medicine.

## General characteristics of the intestinal microbiota in dogs

The canine intestinal microbiota is comprised of a complex microbial population that potentially influences metabolism, immune activity, and the onset of gastrointestinal diseases. Initial studies have revealed that the canine intestinal microbiota is dynamic, with similar bacterial populations present in adjacent intestinal segments, primarily consisting of anaerobic genera. Metagenomic analysis has shown that the dietary nutrient content can modulate bacterial populations and metabolites in the canine intestine. Further research has demonstrated significant correlations between dietary factors and canine gut microbiomes. Additionally, canine gastrointestinal diseases are closely associated with dysbiosis of the intestinal microbiota and metabolic disorders (Huang et al., 2020).

The composition of the intestinal microbiota evolves rapidly during the development of a dog’s life, with the intestine being progressively colonized by crucial bacteria, mainly anaerobic, before reaching adulthood. These bacterial communities are of paramount importance for the host’s health, and any disturbances in their composition can lead to changes in metabolic states, potentially resulting in gastrointestinal disorders (Garrigues et al., 2023).

After birth, the gastrointestinal tract of newborn dogs is rapidly colonized by microorganisms and is highly unstable. In the first two days of life, *Firmicutes* dominate the gastrointestinal tract, representing approximately 60% of bacterial communities (Guard et al., 2017). However, the low microbial abundance and diversity at this stage facilitate potential colonization by external bacteria (Perez-Munoz et al., 2017).

In animal species, the presence of oxygen in the gastrointestinal tract during the first days of life promotes the colonization of obligate and facultative anaerobes (Sanidad and Zeng, 2020). Most *Proteobacteria* and *Bacteroides* fall into these categories and have been shown to be among the first colonizers of the neonatal gut. Oxygen consumption and a decrease in redox potential (which is positive at birth) play a key role in preparing the gut for the subsequent colonization of strict anaerobes, which are necessary for healthy gut function (Moon et al., 2018; Shin et al., 2015; Pereira et al., 2020). Over time, the proportion of aerotolerant bacteria decreases in the puppy’s gut. The increase in abundance of these bacteria in the puppy’s gut is not only related to oxygen homeostasis but also to the ingestion of milk during the neonatal period, as these bacteria can digest milk oligosaccharides and produce lactate (Garrigues et al., 2023).

Significant changes in bacterial populations in the gastrointestinal tract of puppies occur during the first weeks of life, even before they start consuming solid food. These microbial changes, and consequently the biological properties of the microbiome, are mainly induced by neonatal metabolic events such as progressive oxygen consumption in the intestine or the increasing capacity of the intestine to absorb nutrients, produce bile acids, and develop immune functions (Buddington, 2003).

According to Gethings-Behncke et al. (2020), *Proteobacteria* and *Firmicutes* were the most dominant phyla in most rectal samples collected on days 1 and 8 postpartum, confirming their role as the most abundant early colonizers of the intestinal microbiome in canine neonates. The significance of *Enterococcus* in the early rectal microbiome of healthy puppies is still uncertain. While *Enterococcus faecium* is considered a beneficial probiotic in dogs (Subramanian et al., 2015), its increase can lead to changes such as diarrhea and hypocobalaminemia (Shin et al., 2015; Dunn et al., 2017).

According to Beller et al. (2021), in neonates born vaginally, the composition of the meconium microbial population resembles that of the maternal vaginal microbiota, while in neonates born by C-section, it resembles the bacterial composition of the maternal vagina and oral cavity.

Although many studies focus on the microbiota of children, there is still a lack of knowledge about the development of the intestinal microbiota in puppies. Understanding this initial evolution is becoming a fundamental aspect in improving the short and long-term health and well-being of dogs (Garrigues et al., 2023).

## General Characteristics of the Intestinal Microbiota in Humans

Early colonization of the human intestine in newborns begins with exposure to maternal vaginal and fecal microbiota, which is crucial for normal intestinal development and function (Insoft et al., 1996; McCracken and Lorenz, 2001). The impact of microbiome-associated changes during pregnancy on newborns has been extensively studied due to their potential implications for health and disease, both early in life and later on (Nuriel-Ohayon et al., 2016; Vuillermin et al., 2017; Nuriel-Ohayon et al., 2019; Torres et al., 2020). Factors such as inadequate maternal health, cesarean birth, milk quality, antibiotic use, and premature birth have been associated with abnormal development of the neonatal microbiome and potentially linked to various diseases like asthma, atopy, and obesity (Arrieta et al., 2014; Nuriel-Ohayon et al., 2016; Turjeman et al., 2021).

In humans, vaginal delivery leads to a neonatal intestinal microbial population that is dominated by the microbial constituents of the maternal birth canal and feces (e.g., *Lactobacillus* spp. and *Bifidobacterium* spp.), while cesarean section results in a neonatal microbial population dominated by the microbial constituents of the maternal skin (e.g., *Staphylococcus* spp.) (Nuriel-Ohayon et al., 2016). While the adult gut microbiota is mainly composed of bacteria from the *Firmicutes* and *Bacteroidetes* phyla, the neonatal gut microbiota initially consists of microorganisms from the *Proteobacteria* and *Actinobacteria* phyla, which later become more diverse with the emergence of *Firmicutes* and *Bacteroidetes* (Spor et al., 2011; Ottman et al., 2012) (table 1).

**Table 1:** Representation of the main bacterial phyla isolated from humans and dogs.

**PHYLA**	**LOCATION**	**PRESENCE (+), DOMINANCE (++), ABSENCE (0)**	
		**HUMAN**	**DOG**
Firmicutes	Adult intestinal microbiota	+	++
	Newborn	+	++
Bacteroidetes	Adult intestinal microbiota	++	++
	Newborn	0	0
Proteobacteria	Adult intestinal microbiota	+	+
	Newborn	+	+
Actinobacteria	Adult intestinal microbiota	+	+
	Newborn	++	+
Fusobacteria	Adult intestinal microbiota	+	+
	Newborn	0	0

Reference: Coelho et al. 2018; Gethings-Behncke et al., 2020; Ottman et al., 2012; Spor et al., 2011

The predominant bacterial genus in the microbiota of breastfed infants is *Bifidobacterium* (Palmer et al., 2007; Turroni et al., 2012). However, recent studies have also shown a high occurrence of *Enterobacteria* in this population. During the first few weeks, *Proteobacteria*, mainly *Enterobacteriaceae*, dominate the infant intestinal microbiota, while *Bifidobacteria* constitute the second most abundant microbial population, which increases over time along with a decrease in *Enterobacteria* (Arboleya et al., 2015). These early interactions between the microbiota and the host are critical events that support the proper maturation of the human host, leading to the establishment and maintenance of host-microbiota homeostasis during early life, with immediate and long-term implications for health (Milani et al., 2017b).

## Influence of intestinal microbiota on host health

In recent mammal studies, the microbiota has been recognized as a crucial factor in various vital processes of the host, including energy requirements, metabolism, immune activity, and neurobehavioral development. The interaction between the intestinal microbiota, the host, and other somatic cells regulates functions such as digestion, host metabolism, synthesis of vitamins (such as K and B complex), bile acid transformation, proper maturation of gastrointestinal cells, and defense against pathogenic bacteria (Steiner and Ruaux, 2008).

In recent years, the intestinal microbiome has been considered as an organ essential for the host’s survival (Baquero and Nombela, 2012). The gastrointestinal tract’s microbiota is a highly complex structure comprising trillions of microorganisms, depending on the species. For example, there are approximately 10^10^ bacteria in just 1 ml of cow rumen (Matthews et al., 2019), while omnivores like humans and carnivores like dogs have around 10^13^ microorganisms in their guts, predominantly bacteria, but also including viruses and fungi (Gill et al., 2006; Suchodolski, 2011). These microorganisms have a profound symbiotic relationship with their host, providing metabolic capabilities that the host organism alone could not achieve, such as nutrient assimilation and development of the immune system.

The composition of the gastrointestinal microbiota can be influenced by various factors such as age, nutrition, and environment (Tilocca et al., 2017; Hasan and Yang, 2019). Some changes induced by these factors can have beneficial effects on the host’s intestinal health, while others may disrupt the microbial balance (eubiosis) and lead to imbalance (dysbiosis), consequently causing gastrointestinal disturbances or even systemic, metabolic, or autoimmune diseases (Chakraborti, 2015; Tilocca et al., 2017; Nishino et al., 2018; Moffa et al., 2019). Dysbiosis is found in a wide range of diseases, including inflammatory bowel disease (IBD), obesity, allergy, diabetes, and autism (Mondo et al., 2019). A balanced microbial ecosystem is crucial for the host’s health and homeostasis (Guard et al., 2017). Furthermore, during the neonatal and developmental periods, the gut microbiome is even more sensitive to potential disruptors compared to adulthood, and changes in microbiota composition during this period may lead to health disturbances later in life (Han et al., 2018).

One notable example of the gut microbiota’s importance in host health is the existence of a bidirectional communication network between the gut microbiota and the human brain, known as the gut-brain axis. This network involves neural, endocrine, metabolic, and immunological systems/pathways (Heijtz et al., 2011; Ogbonnaya et al., 2015; Lynch and Pedersen, 2016; Fung et al., 2017). Recent studies have investigated how the gut microbiota contributes to central nervous system (CNS) development, including neurogenesis, microglial maturation, and myelination (Erny et al., 2015; Hoban et al., 2016), as well as functions such as cognition, mood, and behavior, including sociability and anxiety (Heijtz et al., 2011; Clarke et al., 2013). Furthermore, the gut microbiota has been shown to play important roles in the pathogenesis and progression of various neurodegenerative disorders, including Parkinson’s disease, Alzheimer’s disease, schizophrenia, autism spectrum disorder, and multiple sclerosis (Martinez et al., 2017; Iannone et al., 2019; Rinninella et al., 2019; Sun et al., 2019; Xue et al., 2020).

Therefore, it appears that the establishment of the intestinal microbiota exerts a crucial influence on the well-being of neonates across various species, impacting vital processes and neurobehavioral development (Steiner and Ruaux, 2008). Consequently, investigating the factors that shape this microbial community emerges as promising research area with the potential to mitigate morbidity and mortality in dogs (Garrigues et al., 2023).

## Influence of mode of delivery on the establishment of neonatal and adult intestinal microbiota and its consequences

Many factors influence the gut microbiota, with age having a significant impact on microbial composition (You and Kim, 2021). The development of the intestinal microbiome begins at birth and undergoes changes in composition throughout the different stages of the host’s life. In human medicine, it has been observed that most intestinal bacterial strains remain stable for decades (Faith et al., 2013). This emphasizes the critical role of early colonization in newborns, as the initial bacteria establish a foundation that can shape the host’s gut functions for a significant portion of its life (Shin et al., 2015; Subramanian et al., 2015). Similarly, Del Carro et al (2022) in their study on canine puppies from birth to the first 60 days of life, observed a progressive reduction in the diversity of isolated bacteria over time, particularly during weaning, when the intestinal microbiota started to resemble that of a young adult condition. Consequently, they emphasized that each mother possesses a unique microbiota profile that significantly influences the composition of the litter’s intestinal microbiota, highlighting the importance of ealy colonization from the maternal side.

Traditionally, it was believed that the gastrointestinal tract of mammals is sterile during intrauterine fetal life, and the inoculation of microorganisms occurs through contact with the mother’s vagina and skin, as well as the ingestion of milk in the first hours after birth (Dunn et al., 2017). This understanding, known as the “sterile uterus paradigm,” has been challenged with the emergence of molecular techniques that enable the detection of bacteria in the placenta, uterus, or amniotic fluid in various mammals. This suggests the transmission of bacteria from mother to fetus within the uterine environment (Aagaard et al., 2014; Wassenaar and Panigrahi, 2014; Alipour et al., 2018).

A study conducted by Zakošek et al. (2020) explored the possibility of intrauterine bacterial colonization in canine fetuses by analyzing the composition of the microbiota in meconium and placenta samples. Bacteria were detected in 86.5% of the meconium samples and 57% of the placenta samples collected immediately after birth. Similar to humans, *Staphylococcus* spp., *Streptococcus* spp., and *Neisseria zoodegmatis*, belonging to the *Firmicutes* and *Proteobacteria* phyla, were the most commonly isolated bacteria in dogs (Dong et al., 2018; He et al., 2020). These findings suggest that *Staphylococcus* is prevalent in the dam’s endometrial microbiota, while *Streptococcus* is more abundant in the vagina. This supports the hypothesis of transplacental transfer of microorganisms, indicating that the meconium of puppies born vaginally partially resembles the microbiota of the dam’s vagina (Lyman et al., 2019). In puppies delivered via cesarean section, the presence of microbiota, primarily *Acinetobacter* spp., *Staphylococci*, and *Bacillus* spp., has been observed in the amniotic fluid and meconium (Rota et al., 2021).

However, it is important to consider that these studies reported low bacterial concentrations using culture-based techniques, which may fail to identify the majority of organisms. Additionally, environmental contamination cannot be completely ruled out when collecting samples from newborns at birth (Gunay et al., 2010; Perez-Munoz et al., 2017).

Conversely, Banchi et al. (2023) sought to investigate the maternal-fetal microbiota in dogs and cats, employing both culture-dependent and culture-independent methods to explore the possibility of in utero colonization. To ensure the reliability of their findings, they rigorously implemented aseptic measures, exclusively including elective cesarean sections performed prior to the onset of the first stage of labor when the cervix remained closed. Moreover, they extended their analysis to include samples from the surgical tray to evaluate potential environmental contamination. The outcomes of this research indicated that bacterial biomass is exceedingly low in healthy full-term canine and feline pregnancies, with evidence pointing to contamination from the mother’s skin as the likely origin of the detected bacteria. However, confirming the presence of viable bacteria in most cases proved challenging, thereby supporting the prevailing “sterile uterus paradigm.” Similarly, Del Carro et al. (2022) also observed a low frequency of isolation of enteric bacteria in the meconium of canine neonates. Despite these results, the topic of intrauterine bacterial transfer remains contentious, prompting the call for future investigations to employ more stringed protocols in verifying and controlling for contamination while also providing insights into bacterial viability (Banchi et al., 2023).

Following birth, the mother’s vertical transfer becomes the first influential factor in modulating the puppy’s intestinal microbiota. Pregnant dogs, for instance, transmit *Bifidobacteria* from their intestinal tract to their offspring (Milani et al., 2017b). In sows, maternal microbial agents from milk, skin, vagina, and feces contribute to approximately 90% of the bacteria in the small intestine of newborns less than 35 days old (Liu et al., 2019). These findings highlight the crucial role of vertical transmission from mother to offspring in shaping the initial composition and diversity of the newborn’s microbiota.

Recent studies in human infants have suggested that the transfer of bacteria from mother to baby is highly dependent on the type of birth, with babies born via cesarean section showing altered microbiota and a higher risk of health problems (Dominguez-Bello et al., 2016; Mortensen et al., 2021). Similar findings have been observed in canine studies, where the meconium of puppies delivered by cesarean section had lower bacterial diversity compared to those born vaginally (Zakošek Pipan et al., 2020; Kajdič et al., 2021). Furthermore, *Staphylococcus* species quickly colonize the meconium of puppies born vaginally, similar to their presence in the mother’s vaginal microbiota (Zakošek Pipan et al., 2020). In human medicine, reduced diversity and early colonization of opportunistic bacteria have been observed in children born via cesarean section, impacting their health during the neonatal period (Dewey et al., 2003; Flaherman et al., 2015). Similarly, puppies born vaginally have been shown to gain weight significantly faster than those born via cesarean section, and the presence of bacterial colonization detected in the meconium correlated with increased weight gain on the third and fourth days of life (Garrigues et al., 2023).

In human studies, babies born vaginally come into contact with maternal vaginal and fecal microbiota, resulting in colonization of the neonatal gut by vaginally associated microorganisms such as *Lactobacillus* and *Prevotella* (Biasucci et al., 2010; Dominguez-Bello et al., 2010). In contrast, babies delivered via cesarean section are not directly exposed to maternal microorganisms and are more likely to be colonized by environmental microorganisms from the mother’s skin, hospital staff, or the hospital environment (Biasucci et al., 2010; Bokulich et al., 2016; Backhed et al., 2015; Rodriguez et al., 2015). Although the differences in microbiota between babies born vaginally and by cesarean section gradually decrease over time, babies delivered via cesarean section tend to maintain a more heterogeneous microbiota up to 12 months of age compared to those born vaginally (Backhed et al., 2015; Martin et al., 2016). Furthermore, persistent differences in the intestinal microbiota between children born via cesarean section and vaginal delivery have been reported at 7 years of age (Salminen et al., 2004; Penders et al., 2006; Neu and Rushing, 2011).

The observed differences in microbiota between babies born vaginally and by cesarean section have been associated with the protective effect of the natural route, as cesarean section has been linked to long-term health implications. Studies have reported a significant reduction in cytokine levels in babies delivered via cesarean section (Malamitsi-Puchner et al., 2005; Jakobsson et al., 2014), as well as an increased risk of immunological disorders such as asthma (Thavagnanam et al., 2008), allergies (Bager et al., 2008), type 1 diabetes (Cardwell et al., 2008), and a higher incidence of obesity (Pei et al., 2014). These findings highlight the importance of the initial intestinal microbiota in the maturation and development of the host’s immune system, emphasizing the impact of the delivery route on the individual’s health throughout adulthood, even though the effects on microbiota composition decrease after the early years of life (Milani et al., 2017b).

## Dogs as a research model for human studies

**Figure 1:**
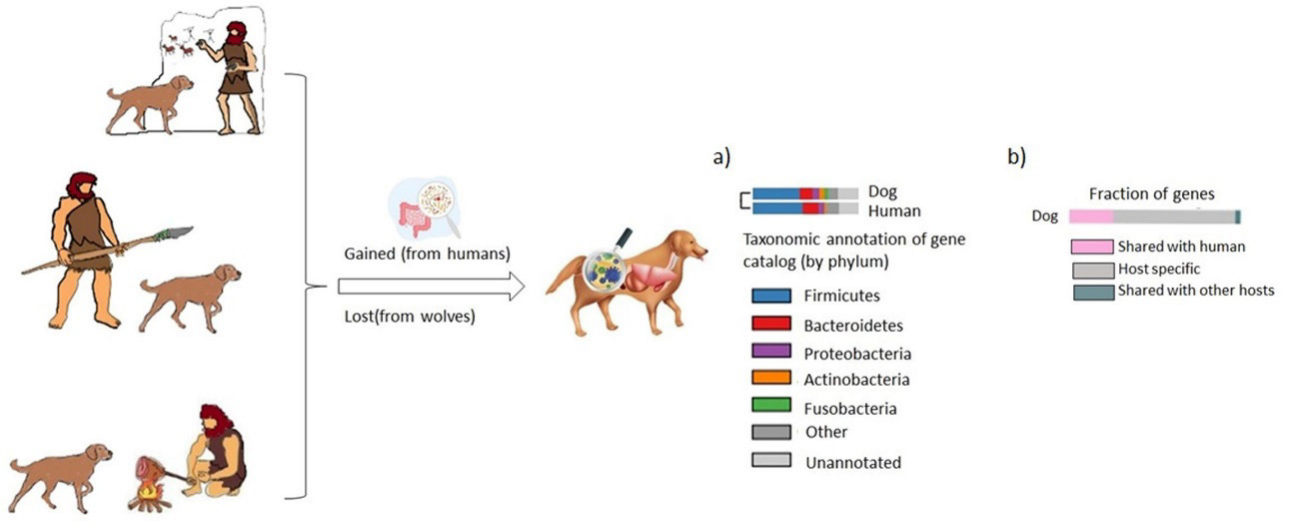
Canine domestication has influenced the composition of the intestinal microbiota. The domestic dog has undergone a loss of specific intestinal bacteria compared to their non-domesticated wolf counterparts, while the close interaction with humans has introduced new gastrointestinal bacteria in dogs. a) Comparison of phylum distribution of genes in the intestinal gene catalogs of domesticated dogs and non-domesticated wolves (b) Representative image showing the proportion of genes shared between the canine intestinal microbiota and the gene catalog of the human intestinal microbiota. (Modified from Coelho et al., 2018).

Recent studies have shown that the canine intestinal microbiota is similar to that of humans (Coelho et al., 2018). This similarity may be attributed, in part, to the domestication of dogs, which has led to changes in their intestinal microbiota compared to non-domesticated wolves. The interaction between dogs and humans has resulted in the loss of certain intestinal bacteria and the emergence of new gastrointestinal bacteria (Coelho et al., 2018) (Fig 1).

Domesticated canines have acquired five bacterial taxa that are also present in the human intestinal microbiota. Additionally, the intestinal microbiota of domesticated canines exhibits greater species diversity when compared to non-domesticated canines. These observations suggest that the canine intestinal microbiota has evolved alongside its host due to cohabitation with humans (Huang et al., 2020). Moreover, canines demonstrate the ability to adapt and develop resilience against dietary changes, a trail also observed in the human intestinal microbiota (Lozupone et al., 2012).

Interestingly, the canine intestinal microbiome globally shows a higher taxonomic and functional overlap with the human intestinal microbiome. Since microbial strains in the intestine are host-specific, this resemblance cannot be solely explained by direct transmission between dogs and humans. Instead, it appears to be a consequence of shared physiology and lifestyle (Coelho et al., 2018).

Natural selection, influenced by similar environmental pressures, may have played a significant role in shaping a shared set of genes between humans and dogs (Coelho et al., 2018). During the process of domestication, genes related to digestion, metabolism, and neurological processes in both species underwent positive selection (Wang et al., 2013; Coelho et al., 2018). These findings suggest a parallel evolution and genetic overlap between humans and dogs in these aspects.

Furthermore, similarities have been observed in the phenotypes of dysbiotic rectal composition in dogs and humans, indicating a potential shared pathophysiology between neonatal diseases in both species associated with early dysbiosis (Tal et al., 2021). Studies have constructed a catalog of genes in the canine intestinal microbiome, revealing that it is predominantly composed of five phyla: *Firmicutes*, *Bacteroidetes*, *Proteobacteria,* *Actinobacteria*, and *Fusobacteria*. The distribution of genetic phyla in the dog intestine is more similar to that of the human intestine, although a higher proportion of Fusobacteria genes is observed (Coelho et al., 2018).

The structural and functional similarity between the canine and human microbiomes suggests that studies conducted in dogs may provide valuable insights into human outcomes. Dogs can serve as models for investigating various diseases, including neurological disorders (Huang et al., 2020). By studying canine intestinal microorganisms, researchers can better understand changes in the canine gut bacteria under different conditions, simulate human diseases using canine models, and explore the intricate interactions between intestinal bacteria and diseases (Huang et al., 2020).

## Vaginal Seeding

Vaginal seeding refers to a technique in which maternal vaginal fluids are used to inoculate a gauze or swab, transferring a portion of the vaginal microbiota to the mouth, nose, or skin of a newborn delivered by cesarean section (Mueller et al., 2015). The aim of vaginal seeding is to facilitate the colonization of the infant’s gut with beneficial bacteria and reduce the subsequent risk of asthma, atopic disease, and immunological disorders associated with cesarean section births (Mueller et al., 2015).

While there has been increasing interest in vaginal seeding, only a few published studies have examined its use in humans (Butler et al., 2021). One small pilot study by Dominguez Bello et al. (2016) found that the microbiomes of babies born via cesarean section who received vaginal seeding were more similar to those born vaginally in terms of their skin, oral cavity, and anal cavity microbiota. However, since no stool samples were collected, the effect of seeding on the intestinal microbiota remains unclear (Butler et al., 2021).

Another study by Song et al. (2021) suggested that vaginal seeding may be associated with the development of an infant’s microbiota during the first year of life, resembling that of vaginally delivered babies more closely than those born via cesarean section. However, further research is needed to determine the potential benefits of this difference and establish a causal relationship between vaginal seeding and subsequent microbiota differences before endorsing it as a routine practice in cesarean sections.

Optimizing the microbiome holds promise as a therapeutic strategy to prevent neonatal diseases associated with dysbiosis, which has significant implications for global health. Vaginal seeding may be a valuable tool in this regard, but further studies are required to fully understand its effects.

## Conclusion

Studying the canine intestinal microbiota offers valuable insights into the intricate interactions between the microorganisms and hosts, shedding light on disease development, progression, and treatment responses. This understanding of the profound impact of the intestinal microbiota on individual health opens up new avenues for translational medicine, enriching healthcare not only for humans but also for domestic animals. Dog, as a research model, hold great promise in bridging the gap between human and animal studies, facilitating the advancement of knowledge and therapeutic approaches in the context of the microbiome. As we unravel the shared complexities of the canine and human microbiotas, we gain a deeper understanding of the fundamental mechanisms that govern health and disease across species, fostering the development of targeted interventions and personalized treatments. Thus, harnessing the potential of canine models in microbiota research paves the way for improved well-being and healthcare outcomes, benefiting both human and canine population alike.

## References

[B001] Aagaard K, Ma J, Antony KM, Ganu R, Petrosino J, Versalovic J (2014). The placenta harbors a unique microbiome. Sci Transl Med.

[B002] Alipour MJ, Jalanka J, Pessa-Morikawa T, Kokkonen T, Satokari R, Hynönen U, Iivanainen A, Niku M (2018). The composition of the perinatal intestinal microbiota in cattle. Sci Rep.

[B003] Arboleya S, Sanchez B, Milani C, Duranti S, Solis G, Fernandez N, Reyes-Gavilan CG, Ventura M, Margolles A, Gueimonde M (2015). Intestinal microbiota development in preterm neonates and effect of perinatal antibiotics. J Pediatr.

[B004] Arrieta MC, Stiemsma LT, Amenyogbe N, Brown EM, Finlay B (2014). The intestinal microbiome in early life: health and disease. Front Immunol.

[B005] Bäckhed F, Roswall J, Peng Y, Feng Q, Jia H, Kovatcheva-Datchary P, Li Y, Xia Y, Xie H, Zhong H, Khan MT, Zhang J, Li J, Xiao L, Al-Aama J, Zhang D, Lee YS, Kotowska D, Colding C, Tremaroli V, Yin Y, Bergman S, Xu X, Madsen L, Kristiansen K, Dahlgren J, Wang J (2015). Dynamics and stabilization of the human gut microbiome during the first year of life. Cell Host Microbe.

[B006] Bager P, Wohlfahrt J, Westergaard T (2008). Caesarean delivery and risk of atopy and allergic disease: meta-analyses. Clin Exp Allergy.

[B007] Banchi P, Colitti B, Del Carro A, Corrò M, Bertero A, Ala U, Del Carro A, Van Soom A, Bertolotti L, Rota A (2023). Challenging the hypothesis of in utero microbiota acquisition in healthy canine and feline pregnancies at term: preliminary data. Vet Sci.

[B008] Baquero F, Nombela C (2012). The microbiome as a human organ. Clin Microbiol Infect.

[B009] Beller L, Deboutte W, Falony G, Vieira-Silva S, Tito RY, Valles-Colomer M, Rymenans L, Jansen D, Van Espen L, Papadaki MI, Shi C, Yinda CK, Zeller M, Faust K, Van Ranst M, Raes J, Matthijnssens J (2021). Successional stages in infant gut microbiota maturation. MBio.

[B010] Biasucci G, Rubini M, Riboni S, Morelli L, Bessi E, Retetangos C (2010). Mode of delivery affects the bacterial community in the newborn gut. Early Hum Dev.

[B011] Bokulich NA, Chung J, Battaglia T, Henderson N, Jay M, Li H, Lieber AD, Wu F, Perez-Perez GI, Chen Y, Schweizer W, Zheng X, Contreras M, Dominguez-Bello MG, Blaser MJ (2016). Antibiotics, birth mode, and diet shape microbiome maturation during early life. Sci Transl Med.

[B012] Buddington RK (2003). Postnatal changes in bacterial populations in the gastrointestinal tract of dogs. Am J Vet Res.

[B013] Butler EM, Reynolds AJ, Derraik JGB, Wilson BC, Cutfield WS, Grigg CP (2021). The views of pregnant women in New Zealand on vaginal seeding: a mixed-methods study. BMC Pregnancy Childbirth.

[B014] Cardwell CR, Stene LC, Joner G, Cinek O, Svensson J, Goldacre MJ, Parslow RC, Pozzilli P, Brigis G, Stoyanov D, Urbonaite B, Sipetic S, Schober E, Ionescu-Tirgoviste C, Devoti G, de Beaufort CE, Buschard K, Patterson CC (2008). Caesarean section is associated with an increased risk of childhood-onset type 1 diabetes mellitus: a meta-analysis of observational studies. Diabetologia.

[B015] Chakraborti CK (2015). New-found link between microbiota and obesity. World J Gastrointest Pathophysiol.

[B016] Chastant-Maillard S, Guillemot C, Feugier A, Mariani C, Grellet A, Mila H (2017). Reproductive performance and pre-weaning mortality: preliminary analysis of 27,221 purebred female dogs and 204,537 puppies in France. Reprod Domest Anim.

[B017] Clarke G, Grenham S, Scully P, Fitzgerald P, Moloney RD, Shanahan F, Dinan TG, Cryan JF (2013). The microbiome-gut-brain axis during early life regulates the hippocampal serotonergic system in a sex-dependent manner. Mol Psychiatry.

[B018] Coelho LP, Kultima JR, Costea PI, Fournier C, Pan Y, Czarnecki-Maulden G, Hayward MR, Forslund SK, Schmidt TSB, Descombes P, Jackson JR, Li Q, Bork P (2018). Similarity of the dog and human gut microbiomes in gene content and response to diet. Microbiome.

[B019] Del Carro A, Corrò M, Bertero A, Colitti B, Banchi P, Bertolotti L, Rota A (2022). The evolution of dam-litter microbial flora from birth to 60 days of age. BMC Vet Res.

[B020] Dewey KG, Nommsen-Rivers LA, Heinig MJ, Cohen RJ (2003). Risk factors for suboptimal infant breastfeeding behavior, delayed onset of lactation, and excess neonatal weight loss. Pediatrics.

[B021] Dong T, Chen T, White RAI, Wang X, Hu W, Liang Y, Zhang Y, Lu C, Chen M, Aase H, Xia Y (2018). Meconium microbiome associates with the development of neonatal jaundice. Clin Transl Gastroenterol.

[B022] Dominguez-Bello MG, De Jesus-Laboy KM, Shen N, Cox LM, Amir A, Gonzalez A, Bokulich NA, Song SJ, Hoashi M, Rivera-Vinas JI, Mendez K, Knight R, Clemente JC (2016). Partial restoration of the microbiota of cesarean-born infants via vaginal microbial transfer. Nat Med.

[B023] Dominguez-Bello MG, Costello EK, Contreras M, Magris M, Hidalgo G, Fierer N, Knight R (2010). Delivery mode shapes the acquisition and structure of the initial microbiota across multiple body habitats in newborns. Proc Natl Acad Sci USA.

[B024] Dunn AB, Jordan S, Baker BJ, Carlson NS (2017). The maternal infant microbiome: considerations for labor and birth. MCN Am J Matern Child Nurs.

[B025] Erny D, Hrabě de Angelis AL, Jaitin D, Wieghofer P, Staszewski O, David E, Keren-Shaul H, Mahlakoiv T, Jakobshagen K, Buch T, Schwierzeck V, Utermohlen O, Chun E, Garrett WS, McCoy KD, Diefenbach A, Staeheli P, Stecher B, Amit I, Prinz M (2015). Host microbiota constantly control maturation and function of microglia in the CNS. Nat Neurosci.

[B026] Faith JJ, Guruge JL, Charbonneau M, Subramanian S, Seedorf H, Goodman AL, Clemente JC, Knight R, Heath AC, Leibel RL, Rosenbaum M, Gordon JI (2013). The long-term stability of the human gut microbiota. Science.

[B027] Flaherman VJ, Schaefer EW, Kuzniewicz MW, Li SX, Walsh EM, Paul IM (2015). Early weight loss nomograms for exclusively breastfed newborns. Pediatrics.

[B028] Fung TC, Olson CA, Hsiao EY (2017). Interactions between the microbiota, immune and nervous systems in health and disease. Nat Neurosci.

[B029] Garrigues Q, Apper E, Rodiles A, Rovere N, Chastant S, Mila H (2023). Composition and evolution of the gut microbiota of growing puppies is impacted by their birth weight. Sci Rep.

[B030] Gethings-Behncke C, Coleman HG, Jordao HWT, Longley DB, Crawford N, Murray LJ, Kunzmann AT (2020). Fusobacterium nucleatum in the colorectum and its association with cancer risk and survival: a systematic review and meta-analysis. Cancer Epidemiol Biomarkers Prev.

[B031] Gill SR, Pop M, DeBoy RT, Eckburg PB, Turnbaugh PJ, Samuel BS, Gordon JI, Relman DA, Fraser-Liggett CMF, Nelson KE (2006). Metagenomic analysis of the human distal gut microbiome. Science.

[B032] Grundy SA (2006). Clinically relevant physiology of the neonate. Vet Clin North Am Small Anim Pract.

[B033] Guard BC, Mila H, Steiner JM, Mariani C, Suchodolski JS, Chastant-Maillard S (2017). Characterization of the fecal microbiome during neonatal and early pediatric development in puppies. PLoS ONE.

[B034] Gunay U, Onat K, Gunay A, Ulgen M (2010). Vaginal, cervical and uterine bacterial flora at the different stages of the reproductive cycle in ovariohysterectomized bitches. J Anim Vet Adv.

[B035] Hasan N, Yang H (2019). Factors affecting the composition of the gut microbiota, and its modulation. PeerJ.

[B036] Han GG, Lee J-Y, Jin G-D, Park J, Choi YH, Kang S-K, Chae BJ, Kim EB, Choi YJ (2018). Tracing of the fecal microbiota of commercial pigs at five growth stages from birth to shipment. Sci Rep.

[B037] He Q, Kwok L-Y, Xi X, Zhong Z, Ma T, Xu H, Meng H, Zhao F, Zhang H (2020). The meconium microbiota shares more features with the amniotic fluid microbiota than the maternal fecal and vaginal microbiota. Gut Microbes.

[B038] Heijtz RD, Wang S, Anuar F, Qian Y, Björkholm B, Samuelsson A, Hibberd ML, Forssberg H, Pettersson S (2011). Normal gut microbiota modulates brain development and behavior. Proc Natl Acad Sci USA.

[B039] Hoban AE, Stilling RM, Ryan FJ, Shanahan F, Dinan TG, Claesson MJ, Clarke G, Cryan JF (2016). Regulation of prefrontal cortex myelination by the microbiota. Transl Psychiatry.

[B040] Huang Z, Pan Z, Yang R, Bi Y, Xiong X (2020). The canine gastrointestinal microbiota: early studies and research frontiers. Gut Microbes.

[B041] Iannone LF, Preda A, Blottière HM, Clarke G, Albani D, Belcastro V, Carotenuto M, Cattaneo A, Citraro R, Ferraris C, Ronchi F, Luongo G, Santocchi E, Guiducci L, Baldelli P, Iannetti P, Pedersen S, Petretto A, Provasi S, Selmer K, Spalice A, Tagliabue A, Verrotti A, Segata N, Zimmermann J, Minetti C, Mainardi P, Giordano C, Sisodiya S, Zara F, Russo E, Striano P (2019). Microbiota-gut brain axis involvement in neuropsychiatric disorders. Expert Rev Neurother.

[B042] Indrebø A, Trangerud C, Moe L (2007). Canine neonatal mortality in four large breeds. Acta Vet Scand.

[B043] Insoft RM, Sanderson IR, Walker WA (1996). Development of imune function in the intestine and its role in neonatal diseases. Pediatr Clin North Am.

[B044] Jakobsson HE, Abrahamsson TR, Jenmalm MC, Harris K, Quince C, Jernberg C, Bjorksten B, Engstrand L, Andersson AF (2014). Decreased gut microbiota diversity, delayed Bacteroidetes colonization, and reduced Th1 responses in infants delivered by caesarean section. Gut.

[B045] Kajdič L, Plavec T, Zdovc I, Kalin A, Zakošek Pipan M (2021). Impact of type of parturition on colostrum microbiota composition and puppy survival. Animals (Basel).

[B046] Konde AM, Gitau GK, Kiptoon J, Gakuya D (2015). Puppy morbidity and mortality among breeding kennels in Nairobi, Kenya. J J Vet Sci Res..

[B047] Liu J, Meng Z, Liu X, Zhang XH (2019). Microbial assembly, interaction, functioning, activity and diversification: a review derived from community compositional data. Mar Life Sci Technol.

[B048] Lourenço MLG, Jericó MM, Neto JPA, Kogika MM (2015). Tratado de Medicina Interna de Cães e Gatos.

[B049] Lozupone CA, Stombaugh JI, Gordon JI, Jansson JK, Knight R (2012). Diversity, stability and resilience of the human gut microbiota. Nature.

[B050] Lynch SV, Pedersen O (2016). The human intestinal microbiome in health and disease. N Engl J Med.

[B051] Lyman CC, Holyoak GR, Meinkoth K, Wieneke X, Chillemi KA, DeSilva U (2019). Canine endometrial and vaginal microbiomes reveal distinct and complex ecosystems. PLoS One.

[B052] Malamitsi-Puchner A, Protonotariou E, Boutsikou T, Makrakis E, Sarandakou A, Creatsas G (2005). The influence of the mode of delivery on circulating cytokine concentrations in the perinatal period. Early Hum Dev.

[B053] Matthews C, Crispie F, Lewis E, Reid M, O’Toole PW, Cotter PD (2019). The rumen microbiome: a crucial consideration when optimising milk and meat production and nitrogen utilisation efficiency. Gut Microbes.

[B054] Martin R, Makino H, Cetinyurek Yavuz A, Ben-Amor K, Roelofs M, Ishikawa E, Kubota H, Swinkels S, Sakai T, Oishi K, Kushiro A, Knol J (2016). Early-life events, including mode of delivery and type of feeding. PLoS One.

[B055] Martinez KB, Leone V, Chang EB (2017). Microbial metabolites in health and disease: navigating the unknown in search of function. J Biol Chem.

[B056] McCracken VJ, Lorenz RG (2001). The gastrointestinal ecosystem: a precarious alliance among epithelium, immunity and microbiota. Cell Microbiol.

[B057] Mila H, Grellet A, Delebarre M, Mariani C, Feugier A, Chastant-Maillard S (2017). Monitoring of the newborn dog and prediction of neonatal mortality. Prev Vet Med.

[B058] Milani C, Duranti S, Bottacini F, Casey E, Turroni F, Mahony J, Belzer C, Delgado Palacio S, Arboleya Montes S, Mancabelli L, Lugli GA, Rodriguez JM, Bode L, de Vos W, Gueimonde M, Margolles A, van Sinderen D, Ventura M (2017). The first microbial colonizers of the human gut: composition, activities, and health implications of the infant gut microbiota. Microbiol Mol Biol Rev.

[B059] Meloni T, Martino PA, Grieco V, Pisu MC, Banco B, Rota A, Veronesi MC (2014). A survey on bacterial involvement in neonatal mortality in dogs. Vet Ital.

[B060] Moon CD, Young W, Maclean PH, Cookson AL, Bermingham EN (2018). Metagenomic insights into the roles of Proteobacteria in the gastrointestinal microbiomes of healthy dogs and cats. MicrobiologyOpen.

[B061] Moffa S, Mezza T, Cefalo CMA, Cinti F, Impronta F, Sorice GP, Santoro A, Di Giuseppe G, Pontecorvi A, Giaccari A (2019). The interplay between immune system and microbiota in diabetes. Mediators Inflamm.

[B062] Mondo E, Marliani G, Accorsi PA, Cocchi M, Di Leone A (2019). Role of gut microbiota in dog and cat’s health and diseases. Open Vet J.

[B063] Mortensen MS, Rasmussen MA, Stokholm J, Brejnrod AD, Balle C, Thorsen J, Krogfelt KA, Bisgaard H, Sorensen SJ (2021). Modeling transfer of vaginal microbiota from mother to infant in early life. eLife.

[B064] Mueller NT, Bakacs E, Combellick J, Grigoryan Z, Dominguez-Bello MG (2015). The infant microbiome development: mom matters. Trends Mol Med.

[B065] Münnich A, Küchenmeister U (2014). Causes, diagnosis and therapy of common diseases in neonatal puppies in the first days of life: cornerstones of practical approach. Reprod Domest Anim.

[B066] Torrazza RM, Neu J (2011). The developing intestinal microbiome and its relationship to health and disease in the neonate. J Perinatol.

[B067] Neu J, Rushing J (2011). Cesarean versus vaginal delivery: long-term infant outcomes and the hygiene hypothesis. Clin Perinatol.

[B068] Nishino K, Nishida A, Inoue R, Kawada Y, Ohno M, Sakai S, Inatomi O, Bamba S, Sugimoto M, Kawahara M, Naito Y, Andoh A (2018). Analysis of endoscopic brush samples identified mucosa-associated dysbiosis in inflammatory bowel disease. J Gastroenterol.

[B069] Nuriel-Ohayon M, Neuman H, Koren O (2016). Microbial changes during pregnancy, birth and infancy. Front Microbiol.

[B070] Nuriel-Ohayon M, Neuman H, Ziv O, Belogolovski A, Barsheshet Y, Bloch N, Uzan A, Lahav R, Peretz A, Frishman S, Hod M, Hadar E, Louzoun Y, Avni O, Koren O (2019). Progesterone increases Bifidobacterium relative abundance during late pregnancy. Cell Rep.

[B071] Ogbonnaya ES, Clarke G, Shanahan F, Dinan TG, Cryan JF, O’Leary OF (2015). Adult hippocampal neurogenesis is regulated by the microbiome. Biol Psychiatry.

[B072] Ottman N, Smidt H, de Vos WM, Belzer C (2012). The function of our microbiota: who is out there and what do they do?. Front Cell Infect Microbiol.

[B073] Palmer C, Bik EM, DiGiulio DB, Relman DA, Brown PO (2007). Development of the human infant intestinal microbiota. PLoS Biol.

[B074] Pei Z, Heinrich J, Fuertes E, Flexeder C, Hoffmann B, Lehmann I, Schaaf B, von Berg A, Koletzko S (2014). Cesarean delivery and risk of childhood obesity. J Pediatr.

[B075] Penders J, Thijs C, Vink C, Stelma FF, Snijders B, Kummeling I, van den Brandt PA, Stobberingh EE (2006). Factors influencing the composition of the intestinal microbiota in early infancy. Pediatrics.

[B076] Pereira AM, Pinna C, Biagi G, Stefanelli C, Maia MRG, Matos E, Segundo MA, Fonseca AJM, Cabrita ARJ (2020). Supplemental selenium source on gut health: insights on fecal microbiome and fermentation products of growing puppies. FEMS Microbiol Ecol.

[B077] Perez-Muñoz M, Arrieta M-C, Ramer-Tait A, Walter J (2017). A critical assessment of the “sterile womb” and “in utero colonization” hypotheses: implications for research on the pioneer infant microbiome. Microbiome.

[B078] Pitlik SD, Koren O (2017). How holobionts get Sick-toward a unifying scheme of disease. Microbiome..

[B079] Rinninella E, Raoul P, Cintoni M, Franceschi F, Miggiano GAD, Gasbarrini A, Mele MC (2019). What Is the healthy gut microbiota composition? A Changing ecosystem across age, environment, diet, and diseases. Microorganisms.

[B080] Rodríguez JM, Murphy K, Stanton C, Ross RP, Kober OI, Juge N, Avershina E, Rudi K, Narbad A, Jenmalm MC, Marchesi JR, Collado MC (2015). The composition of the gut microbiota throughout life, with an emphasis on early life. Microb Ecol Health Dis.

[B081] Rota A, Del Carro A, Bertero A, Del Carro A, Starvaggi Cucuzza A, Banchi P, Corrò M (2021). Does bacteria colonization of canine newborns start in the uterus?. Animals (Basel).

[B082] Salminen S, Gibson GR, McCartney AL, Isolauri E (2004). Influence of mode of delivery on gut microbiota composition in seven-year-old children. Gut.

[B083] Sanidad KZ, Zeng MY (2020). Neonatal gut microbiome and immunity. Curr Opin Microbiol.

[B084] Shao Y, Forster SC, Tsaliki E, Vervier K, Strang A, Simpson N, Kumar N, Stares MD, Rodger A, Brocklehurst P, Field N, Lawley TD (2019). Stunted microbiota and opportunistic pathogen colonization in caesarean-section birth. Nature.

[B085] Shin N-R, Whon TW, Bae J-W (2015). Proteobacteria: microbial signature of dysbiosis in gut microbiota. Trends Biotechnol.

[B086] Song SJ, Wang J, Martino C, Jiang L, Thompson WK, Shenhav L, McDonald D, Marotz C, Harris PR, Hernandez CD, Henderson N, Ackley E, Nardella D, Gillihan C, Montacuti V, Schweizer W, Jay M, Combellick J, Sun H, Garcia-Mantrana I, Gil Raga F, Collado MC, Rivera-Viñas JI, Campos-Rivera M, Ruiz-Calderon JF, Knight R, Dominguez-Bello MG (2021). Naturalization of the microbiota developmental trajectory of Cesarean-born neonates after vaginal seeding. Med..

[B087] Spor A, Koren O, Ley R (2011). Unravelling the effects of the environment and host genotype on the gut microbiome. Nat Rev Microbiol.

[B088] Steiner JM, Ruaux CG (2008). Small animal gastroenterol..

[B089] Subramanian S, Blanton LV, Frese SA, Charbonneau M, Mills DA, Gordon JI (2015). Cultivating healthy growth and nutrition through the gut microbiota. Cell.

[B090] Suchodolski JS (2011). Intestinal microbiota of dogs and cats: a bigger world than we thought. Vet Clin North Am Small Anim Pract.

[B091] Sun J, Xu J, Ling Y, Wang F, Gong T, Yang C, Ye S, Ye K, Wei D, Song Z, Chen D, Liu J (2019). Fecal microbiota transplantation alleviated Alzheimer’s disease-like pathogenesis in APP/PS1 transgenic mice. Transl Psychiatry.

[B092] Tal S, Tikhonov E, Aroch I, Hefetz L, Turjeman S, Koren O, Kuzi S (2021). Developmental intestinal microbiome alterations in canine fading puppy syndrome: a prospective observational study. NPJ Biofilms Microbiomes.

[B093] Tilocca B, Burbach K, Heyer CME, Hoelzle LE, Mosenthin R, Stefanski V, Camarinha-Silva A, Seifert J (2017). Dietary changes in nutritional studies shape the structural and functional composition of the pigs’ fecal microbiome: from days to weeks. Microbiome.

[B094] Thavagnanam S, Fleming J, Bromley A, Shields MD, Cardwell CR (2008). A meta-analysis of the association between Caesarean section and childhood asthma. Clin Exp Allergy.

[B095] Torres J, Hu J, Seki A, Eisele C, Nair N, Huang R, Tarassishin L, Jharap B, Cote-Daigneault J, Mao Q, Mogno I, Britton GJ, Uzzan M, Chen CL, Kornbluth A, George J, Legnani P, Maser E, Loudon H, Stone J, Dubinsky M, Faith JJ, Clemente JC, Mehandru S, Colombel JF, Peter I (2020). Infants born to mothers with IBD present with altered gut microbiome that transfers abnormalities of the adaptative imune system to germ-free mice. Gut.

[B096] Turjeman S, Collado MC, Koren O (2021). The gut microbiome in pregnancy and pregnancy complications. Curr Opin Endocr Metab Res.

[B097] Turroni F, Peano C, Pass DA, Foroni E, Severgnini M, Claesson MJ, Kerr C, Hourihane J, Murray D, Fuligni F, Gueimonde M, Margolles A, De Bellis G, O’Toole PW, van Sinderen D, Marchesi JR, Ventura M (2012). Diversity of bifidobacteria within the infant gut microbiota. PLoS One.

[B098] Vannucchi CI, Silva LCG, Lúcio CF, Regazzi FM, Veiga GAL, Angrimani DSR (2012). Prenatal and neonatal adaptations with a focus on the respiratory system. Reprod Domest Anim.

[B099] Vassalo FG, Simões CRB, Sudano MJ, Prestes N, Lopes MD, Chiacchio SB, Lourenço MLG (2015). Topics in the routine assessment of newborn puppy viability. Top Companion Anim Med.

[B100] Vuillermin PJ, Macia L, Nanan R, Tang MLK, Collier F, Brix S (2017). The maternal microbiome during pregnancy and allergic disease in offspring. Semin Immunopathol.

[B101] Wang GD, Zhai W, Yang HC, Fan RX, Cao X, Zhong L, Wang L, Liu F, Wu H, Cheng L-G, Poyarkov AD, Poyarkov NA, Tang SS, Zhao WM, Gao Y, Lv XM, Irwin DM, Savolainen P, Wu CI, Zhang YP (2013). The genomics of selection in dogs and the parallel evolution between dogs and humans. Nat Commun.

[B102] Wassenaar TM, Panigrahi P (2014). Is a foetus developing in a sterile environment?. Lett Appl Microbiol.

[B103] Xue LJ, Yang XZ, Tong Q, Shen P, Ma SJ, Wu SN, Zheng ZL, Wang HG (2020). Fecal microbiota transplantation therapy for Parkinson’s disease: a preliminary study. Medicine (Baltimore).

[B104] You I, Kim MJ (2021). Comparison of gut microbiota of 96 healthy dogs by individual traits: breed, age, and body condition score. Animals (Basel).

[B105] Zachariassen LF, Krych L, Rasmussen SH, Nielsen DS, Kot W, Holm TL, Hansen AK, Hansen CHF (2019). Cesarean section induces microbiota-regulated immune disturbances in C57BL/6 mice. J Immunol.

[B106] Zakošek Pipan M, Kajdic L, Kalin A, Plavec T, Zdovc I (2020). Do newborn puppies have their own microbiota at birth? Influence of type of birth on newborn puppy microbiota. Theriogenology.

